# Can concurrent core biopsy and fine needle aspiration biopsy improve the false negative rate of sonographically detectable breast lesions?

**DOI:** 10.1186/1471-2407-10-371

**Published:** 2010-07-16

**Authors:** Yao-Lung Kuo, Tsai-Wang Chang

**Affiliations:** 1 Department of Surgery, National Cheng Kung University Hospital, College of Medicine, National Cheng Kung University, Tainan and Dou-Liou Branch, Taiwan

## Abstract

**Background:**

The aims of this study were to determine the accuracy of concurrent core needle biopsy (CNB) and fine needle aspiration biopsy (FNAB) for breast lesions and to estimate the false-negative rate using the two methods combined.

**Methods:**

Over a seven-year period, 2053 patients with sonographically detectable breast lesions underwent concurrent ultrasound-guided CNB and FNAB. The sonographic and histopathological findings were classified into four categories: benign, indeterminate, suspicious, and malignant. The histopathological findings were compared with the definitive excision pathology results. Patients with benign core biopsies underwent a detailed review to determine the false-negative rate. The correlations between the ultrasonography, FNAB, and CNB were determined.

**Results:**

Eight hundred eighty patients were diagnosed with malignant disease, and of these, 23 (2.5%) diagnoses were found to be false-negative after core biopsy. After an intensive review of discordant FNAB results, the final false-negative rate was reduced to 1.1% (*p-value = *0.025). The kappa coefficients for correlations between methods were 0.304 (*p-value *< 0.0001) for ultrasound and FNAB, 0.254 (*p-value *< 0.0001) for ultrasound and CNB, and 0.726 (*p-value *< 0.0001) for FNAB and CNB.

**Conclusions:**

Concurrent CNB and FNAB under ultrasound guidance can provide accurate preoperative diagnosis of breast lesions and provide important information for appropriate treatment. Identification of discordant results using careful radiological-histopathological correlation can reduce the false-negative rate.

## Background

Breast cancer has become a serious threat to women's health in Taiwan over recent decades. Breast cancer ranks fourth among the top 10 causes of death from cancer in women, and the death rate has increased from 5 to 12.8 per 100,000 population in the past two decades (data from the Bureau of Health Promotion, Department of Health, Taiwan; http://www.doh.gov.tw/statistic/data). Because of this increase in the death rate, screening has become more important in health care in Taiwan, and screening programs with mammography and ultrasound (US) are used routinely. However, as in several Asian countries, Taiwanese women have smaller breasts and denser breast tissue than do Western women,[1,2] and this can cause false-negative findings on mammography. Breast US is an excellent diagnostic method for efficiently detecting breast tumors in dense breast tissue[3-5].

Breast lesions detected in a screening US should be diagnosed histopathologically because any misdiagnosis can delay treatment of the cancer. Minimizing the number of unnecessary surgeries is essential in the diagnosis and treatment of breast tumors. Three main diagnostic procedures are used in the pathological examination of suspicious breast lesions: fine needle aspiration biopsy (FNAB), core needle biopsy (CNB), and surgical open biopsy. FNAB and CNB are minimally invasive procedures that can be performed on an outpatient basis[6,7].

The diagnostic accuracies of CNB and FNAB have been compared. FNAB is a relevant test, especially in combination with palpation and imaging findings[8-10]. Compared with histological evaluation, CNB is generally considered to produce better results than the cytological results acquired by FNAB,[11-15] especially for lesions highly suspected of being malignant. However, a CNB can produce false-negative results because of missampling or technical failure. Both FNAB and CNB have a specificity approaching 100% in the presence of carcinoma, but their sensitivities range between 80% and 97%. The overall sensitivity may increase when the tests are combined[6,7,14,15,17].

In this investigation, we analyzed data derived from a combination of FNAB and CNB under US guidance. The objectives of this study were to confirm the diagnostic accuracy of concurrent US-guided FNAB and CNB and to analyze the methodology to identify and reduce the rate of false-negative diagnoses.

## Methods

### Study population

From April 2000 to June 2007, the study population of women with breast abnormalities who presented with ultrasonically visible lesions was evaluated at National Cheng Kung University Hospital, Tainan, Taiwan, using US-guided FNAB and CNB. A total of 2053 patients (age range, 12-102 years; mean age, 44.8 years) were identified. No initial biopsies by surgical excision, stereotactic biopsy, or any other studies of these lesions were performed before the US-guided biopsies. Patients with lesions not clearly visualized on US were excluded from this study. Ethical approval was provided by Human Experiment and Ethics committee of the National Cheng Kung University Hospital (ER-99-074).

Concurrent US-guided CNB and FNAB were used to evaluate all sonographically visible lesions and were performed in the supine position with the arm elevated, using a high-resolution 10-14-MHz linear array transducer with adjustable puncture and biopsy guides (Falcon Premium 2101, B-K Medical's, Herlev, Denmark). All procedures were performed under local anesthesia via an injection of 5 cc of 1% lidocaine into the skin and subcutaneous tissue, and around the tumor. For FNAB, the specimen was taken with at least 10 passes without needle withdrawal and under constant negative pressure. The cytology was checked immediately under a microscope in the outpatient clinic to ensure that examinable cells were obtained. For CNB, a 14-gauge automated needle device with a 22 mm throw biopsy gun (Bard-Magnum Biopsy Instrument, Covington, GA, USA) was used. The needle was placed at the edge of the lesion in the prefiring position, and the 22 mm core needle throw was executed. The passage of the needle across the index lesion was confirmed under direct visualization with postfire US images. At least five specimens were obtained from each lesion. Specimens were placed in formalin and then submitted for histopathological evaluation; the number of samples was dependent on the lesion size, consistency, and ultrasonographic visibility. Patients with discordance between the core biopsy, fine needle biopsy, and US findings were reviewed, and if necessary, another biopsy or further excision was performed.

The records of all pathologic breast reports were collected and classified under the following categories[18].

B: Benign

I: Benign, but uncertain malignant potential

S: Suspicious of malignancy

M: Malignant

The findings of all breast US examinations were classified after the modification according to American College of Radiology Breast Imaging Reporting and Data System Atlas for Ultrasound[19] as follows.

B: Benign (BI-RADS II)

I: Probably benign findings (BI-RADS III)

S: Suspicion of malignancy (BI-RADS IV)

M: Highly suggestive of malignancy (BI-RADS V)

For patients with lesions with a benign finding after the rebiopsy, a sonography follow-up at 6 months was recommended. For evaluation of false-negative cases, the case series were reviewed through linkage with the National Cancer Registry of Taiwan.

### Statistical analysis

After tabulation of the data, the specificity, sensitivity, negative and positive predictive values, false-negative rate, false-positive rate, and accuracy were determined for the types of lesion to calculate whether the concurrent CNB and FNAC results agreed with the histopathological findings of the final excisional biopsy, surgery, or clinical follow-up. Chi-squared test assessed paired observation on two variables, which independent of each other. Kappa coefficient measures the agreement between the binary variable. All *p*-values were two-tailed, with *p = *0.05 or lower considered significant. Statistical analysis was performed using SPSS for Windows, version 13.0 (Chicago, IL).

## Results

CNB and FNAB under US guidance were performed concurrently to assess the breast lesions by ascertaining a histological diagnosis. A total of 2053 CNB and FNAB were performed. Of these, 880 patients were diagnosed with malignant disease. On US, the average size of the lesion was 16.7 mm (median 13 mm; range 4-150 mm). The sensitivity of CNB and FNAB under US guidance to identify infiltrating breast lesions was 98% and 95%, and the specificity was 99% and 86%, respectively. The correlation between US and FNAB was 306.775 (*p *< 0.0001) on the chi-squared test, and the kappa coefficient was 0.304 (*p *< 0.0001). The sensitivity and specificity were 27.07% and 97.91%, respectively. The positive predictive value was 86.12%, and the negative predictive value was 73.7% (Table 1). The correlation between US and CNB was 289.732 (*p *< 0.0001) on the chi-squared test, and the kappa coefficient was 0.254 (*p *< 0.0001). The sensitivity and specificity were 23.3% and 99.66%, respectively. The positive predictive value was 98.09%, and the negative predictive value was 63.39% (Table 2). The correlation between FNAB and CNB was 1137.806 (*p *< 0.0001) on the chi-squared test, and the kappa coefficient was 0.726 (*p *< 0.0001). The sensitivity and specificity were 72.61% and 97.78%, respectively. The positive predictive value was 96.09%, and the negative predictive value was 82.64% (Table 3).

**Table 1:** Correlation between ultrasound and FNAB

		FNAB		Total
			
		*M*	*B, I, S*	
**Ultrasound**	***M***	180	29	209
	***B, I, S***	485	1359	1844

Total		665	1388	2053

Pearson chi-squared	*p*-value	Kappa coefficient		*p*-value

306.775	*< 0.0001*	0.304		*< 0.0001*

Sensitivity	0.2707	Positive predictive value		0.8612
Specificity	0.9791	Negative predictive value		0.7370

B: benign, I: indeterminate, S: suspicious, M: malignant

**Table 2:** Correlation between ultrasound and CNB

		Core biopsy		Total
			
		*M*	*B, I, S*	
**Ultrasound**	***M***	205	4	209
	***B, I, S***	675	1169	1844

Total		880	1173	2053

Pearson chi-squared	*p*-value	Kappa coefficient		*p*-value

289.732	*< 0.0001*	0.254		*< 0.0001*

Sensitivity	0.2330	Positive predictive value		0.9809
Specificity	0.9966	Negative predictive value		0.6339

B: benign, I: indeterminate, S: suspicious, M: malignant

**Table 3:** Correlation between FNAB and CNB

		Core biopsy		Total
			
		*M*	*B, I, S*	
**FNAB**	***M***	639	26	665
	***B, I, S***	241	1147	1388

Total		880	1173	2053
Pearson chi-squared	*p*-value	Kappa coefficient		*p*-value

1137.806	*< 0.0001*	0.726		*< 0.0001*

Sensitivity	0.7261	Positive predictive value		0.9609
Specificity	0.9778	Negative predictive value		0.8264

B: benign, I: indeterminate, S: suspicious, M: malignant

### Clinical features of false-negative CNB findings

Overall, 23 patients had an initial benign CNB and a subsequent diagnosis of malignancy, giving a false-negative rate of 2.5%. The distribution of the pathology of these cases was as follows: intraductal papilloma (n = 6), fibrocystic change (n = 5), atypical ductal hyperplasia (n = 4), fibrous mastopathy (n = 2), papillary lesion (n = 1), sclerosing adenosis (n = 1), lactating adenosis (n = 1), atypical cell (n = 1), chronic inflammation (n = 1), and other (n = 1) (Table 4).

**Table 4:** Distribution of the cytological and pathological results from the 23 false-negative cases

Patient Nr.	U	FNAB	U/F	CNB	Final Pathology	
1	S	M	SM	ADH	DCIS	*

2	S	S	SS	Papilloma	IDC	

3	I	I	II	Fibrocystic change	DCIS	

4	B	M	BM	Fibrocystic change	IDC	*

5	S	M	SM	ADH	Apocrine CA	*

6	I	M	IM	Fibroadipose tissue	IDC	*

7	M	M	MM	Lactating adenosis	IDC	*

8	I	I	II	Papilloma	DCIS	

9	I	M	IM	Papilloma	IPC	*

10	I	I	II	Papilloma	IDC	

11	S	M	SM	Atypical cell	IDC	

12	S	M	SM	Fibrocystic change	IDC	*

13	S	M	SM	Sclerosing adenosis	IDC	*

14	S	M	SM	Chronic inflammation	IDC	*

15	M	I	MI	Fibrocystic change	IDC	

16	S	M	SM	ADH	IDC	*

17	I	I	II	Papilloma	IDC	

18	I	I	II	Papilloma	DCIS	

19	I	M	IM	Fibrous mastopathy	DCIS	*

20	I	M	IM	No evidence of malignancy	Mucinous carcinoma	*

21	S	M	SM	ADH	DCIS	

22	S	M	SM	Papillary lesion	Mucinous CA	*

23	I	I	II	Fibrocystic change	IDC	

* Cases detected by fine needle aspiration biopsy

U: Ultrasound

U/F: Ultrasound/fine needle aspiration biopsy

B: Benign, I: Benign, but uncertain malignant potential, S: Suspicious of malignancy, M: Malignant, ADH: atypical ductal hyperplasia, DCIS: ductal carcinoma in situ, IDC: invasive ductal carcinoma, IPC: invasive papillary carcinoma

### Correlation between the FNAB and CNB findings

In 13 of the 23 false-negative cases, malignancy was shown in the FNAB (adenocarcinoma in patients 1, 4-7, 9, 12-14, 16, 19, 20, and 22) (Table 4). Using this information, the number of false-negative cases could be reduced to 10, which produced a false-negative rate of 1.1%.

Compared with the rate of 2.5% obtained by CNB only, concurrent examination with CNB and FNAB reduced the false-negative rate by 44% (2.5% to 1.1%, p = 0.025).

In these 23 false-negative cases, discordance was recognized immediately in nine because the CNB diagnosis was either papilloma (patients 2, 8, 10, 17, and 18) or atypia (patient 11), and there was a malignant appearance in one patient on sonography (patient 15), which required an open biopsy. In two cases (patients 3 and 21), microcalcification was recognized on mammography one year later, and patient 23 showed progression at the annual follow-up.

## Discussion

Accurate preoperative assessment of breast lesions is crucial for treatment planning. In particular, image-guided biopsy has become an established technique. Information on the best biopsy modality to secure a diagnosis of breast lesions is controversial. CNB has been shown to be an excellent tool when working with true tissue specimens because it permits the evaluation of both the architectural and cytological patterns. The diagnostic accuracy of routine paraffin-embedded CNB samples has been verified since the early 1990s, and in their review article, Usami *et al *reported high concordance between the diagnoses from CNB and surgical biopsy[20]. The study by Dillon *et al *of 2427 core biopsies taken using three different CNB modalities had an overall false-negative rate of 6.1%. However, US-guided CNB showed the lowest false-negative rate of 1.7%[21]. The accuracy of CNB is associated with the number of CNBs taken. In a prospective study of FNAC and CNB in the diagnosis of breast cancer in 143 patients with a palpable lump measuring > 2 cm,[22] four core biopsy specimens were taken with a 14-gauge 10 cm biopsy needle using an automated spring-loaded device. The sensitivity of CNB increased with the number of cores taken (one core, 76.2%; two cores, 80.9%; three cores, 89.2%; four cores, 95.2%)[6]. To ensure accuracy, at least five cores were taken from each of our patients.

FNAB is a simple, quick, and relatively painless procedure. However, the false-negative rate in the presence of cancer is 6-11%[8,23]. Factors that may influence these results include the experience of the clinician and pathologist, and the size and histological type of the tumor[7,24]. Inadequate sampling is a contributory factor to the reduced sensitivity of cytology. This can be improved by instant cytodiagnosis after sampling in the outpatient clinic, which can also confirm the presence of examinable cells. If the examined cells are absent, the sampling will repeat immediately in the outpatient clinic. Nevertheless, the reading of the CNB slides is only possible after formalin fixation by the pathologist.

For the diagnosis of sonographically detectable breast lesions, US-guided FNAB was performed as the initial sampling method, and only for uncertain lesions, a complementary CNB was performed at our facility. This study provides data on US-guided biopsy, particularly in relation to the proportion of breast cancer cases in the study population. US-guided techniques are performed in real time, and the direct visualization of needle placement with US allows the accuracy of the sampling to be assessed. The procedure time is also shorter. Therefore, US-guided biopsies have considerable advantages over stereotactic techniques. US-guided biopsy causes less patient discomfort compared with a stereotactic-guided biopsy because the patient is in a prone position, and it does not involve ionizing radiation and is less expensive than stereotactic techniques[8,25-27]. False-negative rates of 0.6-22.2% have been reported [11,22,26,28-46]. Only a few studies have been published on sonographic guidance [26,28,32,43,46]. To our knowledge, our study involves one of the largest sample sizes studied to investigate US-guided biopsy and demonstrates that the combination of FNAB and CNB is accurate with a false-negative rate of 1.1%.

A false-negative diagnosis may delay the treatment of breast cancer. The analysis by Dillon *et al *of the management and outcome of patients with false-negative cores showed that reviewing the radiological, clinical, and pathological results after the biopsy reduces the delay in the cancer diagnosis to less than one month[21]. US and clinical findings were found to raise the level of suspicion in most of these cases, and FNAB can help the clinician recognize suspicious lesions. In 13 of the 23 patients with false-negative biopsies in our study, it was primarily the FNAB findings that prompted further investigation. This demonstrates a benefit of FNAB because it decreased the false-negative rate by 44% in patients who had this procedure. Combining CNB and FNAB techniques improves the sensitivity of the diagnostic procedure and is supported by other studies[7,13,15,17,47].

FNAB and CNB are complementary in the accurate diagnosis of breast cancer. There is a small risk of misdiagnosis, which was shown by the need for an open surgical approach in nine patients. Concurrent CNB and FNAB as routine assessments could have reduced the false-negative rate in our study from 2.5% (23 of 903) to 1.1% (10 of 903). Thirteen invasive cancers were found by FNAB after being proved by open biopsy despite a benign diagnosis after CNB. Because the cytological finding did not agree with the suspicious FNAB results, 13 patients were shown to have invasive cancers. After subtracting these cases, only 10 cases of true core misses would have had a false-negative diagnosis and resulted in the subsequent suboptimal treatment of patients. Fortunately, one of these lesions was removed at the patient's request. Therefore, if a discordant benign lesion in a core biopsy is recognized promptly by FNAB, a re-biopsy is warranted, so that a false-negative diagnosis can be identified and prevented. To identify the discordance between imaging, FNAB, and CNB, the breast specialist must be familiar with the sonographic features and the histopathological details, and be able to correlate these data. Our classification using four categories can simplify the diagnosis and thus improve the detection of false-negative cases.

High-risk lesions that have uncertain malignant potential or are suspicious for malignancy, such as atypical ductal hyperplasia, papillary lesions, or fibrocystic changes with atypical features, can cause the underestimation of carcinoma [48-53]. Some authors have suggested using a second biopsy or open biopsy in cases of imaging-histological discordance[54,55]. There is a lack of clarity regarding the optimal management of these lesions. Our study shows that FNAB might reduce the need for an unnecessary open biopsy of these lesions. However, papillary lesions can cause underestimation of breast carcinoma[56]. In some institutions, surgical excision is performed for papillary lesions[57-60]. In a review of 57 patients with different papillary subtypes, Sydnor showed an incidence of carcinoma in benign papilloma of 3% compared with 67% for atypical papilloma[61]. This demonstrates the wide spectrum of papillary lesions and the indications for surgical excision. Papillary lesions tend to present with intralesional heterogeneity, and there is a risk of concurrent or subsequent malignancy.

## Conclusion

This study has demonstrated that concurrent US-guided CNB and fine needle biopsy are accurate for the histological diagnosis of breast tumors. The combination of histopathological and radiological findings can provide important information for the prompt recognition of the discordant results in the one-stop breast outpatient clinic. Using a combined US, FNAB, and CNB assessment and review could minimize the delay in the diagnosis of breast cancer in women with false-negative core biopsies.

## Competing interests

The authors declare that they have no competing interests.

## Authors' contributions

CTW designed the concept of this study, drafted the manuscript and performed treatment. CTW collected the data. KYL performed the statistical analysis. KYL designed the concept of this study and provided treatment coordination. All authors read and approved the final manuscript.

## Pre-publication history

The pre-publication history for this paper can be accessed here:

http://www.biomedcentral.com/1471-2407/10/371/prepub
